# Descriptive Study of Oral Health in an Indigenous Child Population of Baka Pygmies in Cameroon

**DOI:** 10.3390/dj11100237

**Published:** 2023-10-12

**Authors:** Nicias Afoumpam Poni, David Ribas-Pérez, Javier Flores-Fraile, Paloma Villalva Hernández-Franch, Diego Rodríguez-Menacho, Antonio Castaño-Séiquer

**Affiliations:** 1 Odontología Social Luis Seiquer Foundation, 41001 Sevilla, Spain; niciasponi@gmail.com; 2 Department of Stomatology, University of Seville, 41001 Seville, Spain; drmenacho@us.es (D.R.-M.); acastano@us.es (A.C.-S.); 3 Department of Surgery, University of Salamanca, 37002 Salamanca, Spain; j.flores@usal.es; 4 Andalusian Health Service, 41001 Seville, Spain; paloma.villalva.sspa@juntadeandalucia.es

**Keywords:** oral health, epidemiology, indigenous people, Baka pygmies, Cameroon

## Abstract

Indigenous populations around the world experience a disproportionate burden of oral diseases and health conditions. These inequalities are likely due to a complex web of socioeconomic, cultural, and health determinants. The Baka pygmies of southern Cameroon find themselves in this context of an indigenous population with health inequities. The purpose of this study was to describe the oral health status, diet, hygiene habits, and access to health services of the Baka pygmy children, from which different care needs will emerge in order to develop health strategies. A descriptive cross-sectional study was conducted in 22 Baka pygmy camps randomly selected. The study population consisted of children aged 5–6 years and 11–12 years chosen by a consecutive sampling technique. The examination was performed using a data sheet based on World Health Organization (WHO) criteria and recommendations which consisted of an oral health questionnaire for children and an oral health assessment form for children. A total of 120 children participated in the study. Extraoral examination of the study population revealed the presence of noma (1%) in the age range of 5–6 years. A total of 2713 teeth were examined, and the DMFT/dft index of the sample was 0.71 with a predominant caries component. The periodontal status showed 87% bleeding on probing. Seven percent of the sample presented a need for immediate urgent treatment due to pain and/or infection. Eighty-seven percent of the sample reported never having been examined by a dentist. The examination and oral care they received was only from nongovernmental organizations (NGOs). The conclusion of this descriptive study is that the precarious oral health situation of pygmy children combined with the absence of care services in general for the Baka pygmies generate a situation of great vulnerability.

## 1. Introduction

Oral diseases are a major public health problem due to their high prevalence and incidence in all regions of the world, and, as with many diseases, they mainly affect disadvantaged and socially marginalized populations such as the indigenous population in general [1,2]. The most severe forms of oral diseases such as noma, cancers of the oral cavity, ulcerative necrotic gingivitis, and cleft lip are observed more in countries affected by poverty, precariousness, and malnutrition. Moreover, in these populations there are marked deficiencies in oral health care services, which represents a real public health problem in many African countries. In addition to all this is the psychological and social impact of these diseases that significantly reduce the quality of life of the individuals who suffer from them [1,3].

Untreated dental caries has a very high prevalence. According to the WHO, it affects 60% to 90% of school-age children and the vast majority of adults. Worldwide, data on carious tooth decay rates show that developed countries have a much lower rate than developing countries [1,4,5]. More specifically in Africa, the development of cities is linked to an increase in the consumption of a diet containing sugary foods and, as a consequence, an increase in the prevalence of dental caries. Added to this is the fact that around 90% of these cases of decayed teeth are not treated and generally receive treatment when the process is already at a very advanced stage where the only option is to extract the affected tooth [5,6,7].

According to the WHO, noma is the most severe oral disease on the African continent, occurring in more than 39 countries in Africa with an incidence of 20 cases per 100,000 people, affecting more children aged 2 to 6 years, and is usually found in contexts of extreme poverty, poor oral hygiene, viral infection, and above all chronic malnutrition and inadequate health care. Noma is a type of gangrene that destroys the mucous membranes of the mouth and other tissues. It occurs in malnourished children living in areas of poor hygiene [1,3,8,9]. In addition, the course of this disease leaves much wear and tear at the dental, bone, and craniofacial levels and is psychologically harmful, and the treatment is very complex, interdisciplinary, and difficult to approach in certain social and health contexts [10,11,12].

The second most severe oral disease in Africa after noma is necrotizing gingivitis [3,8]. The risk factors for this lesion are very similar to those of noma including poor oral hygiene, malnutrition, and systemic and viral diseases [13,14,15,16].

### 1.1. Oral Health in Cameroon

In Cameroon, data on the oral health of children in some regions show a prevalence of 66.9% for caries and 60.7% for gingivitis in the South [17]. In the West, a study presents a prevalence of 78% for gingivitis, 39% for periodontitis, and 30% for dental caries and concludes that the main barriers to access to health services were economic (67.8%) and the distance to health care [18]. In the Center, a study of a rural population aged 5 to 17 years revealed that 21% had caries, 44.8% had gingivitis, 52% had dental fluorosis, 53.5% had enamel erosion, 32.8% had lesions of the oral mucosa, and 68.8% of the rural population had mouths in a pathological state with an urgent need for dental care [19].

### 1.2. The Baka Pygmies

The first inhabitants of the Cameroonian forest, the Baka pygmies, who are part of the indigenous peoples found in Cameroon, are characterized by an average male height of less than 155 cm, a hunter–gatherer lifestyle, and practices such as the use of plants and plant fibers for therapeutic purposes and also dental mutilation, which, as in several equatorial African countries, remains a technically traumatic act [13,20,21]. 

However, the Baka pygmies, like most of the world’s indigenous populations, face many problems such as social exclusion, isolation, malnutrition, low economic and schooling levels, environmental problems due to deforestation, poor health conditions, and inequality in terms of access to and availability of health care resources [2,22,23,24].

In this regard, the Baka pygmies only benefit from the social, health, and educational support of certain nongovernmental organizations (NGOs). All these conditions led us to take an interest in the oral health of this indigenous population, and the objective of this study was to describe the oral health status, diet, hygiene habits, and access to health services of Baka pygmy children in southern Cameroon in order to develop health strategies adapted to this context.

## 2. Materials and Methods

### 2.1. Design and Sampling

**Figure 1:**
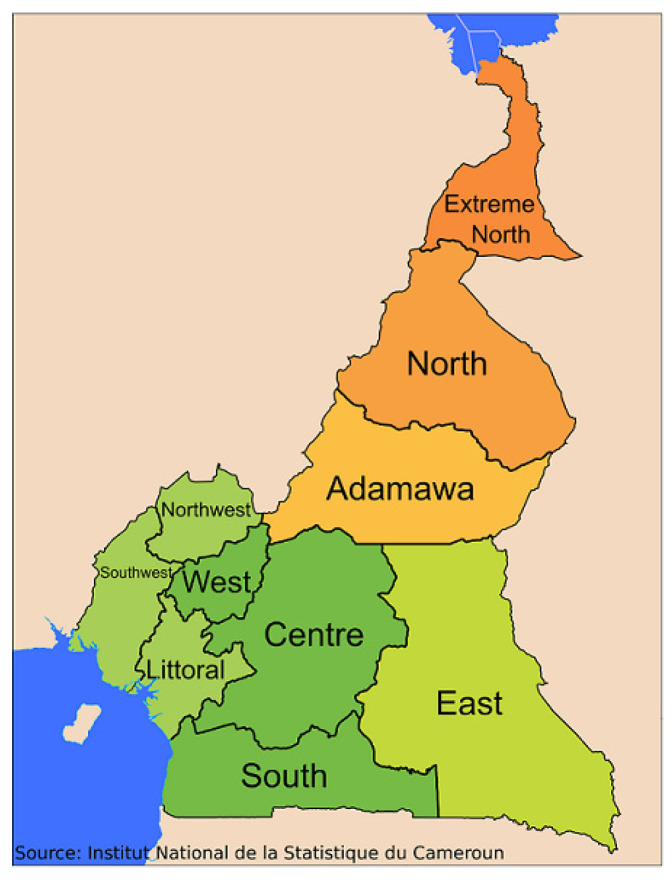
Regional distribution of Cameroon. Source: National Institute of Statistics of Cameroon. https://ins-cameroun.cm/en/ (accessed on 18 July 2023).

A descriptive and observational cross-sectional study was conducted in 22 Baka camps randomly selected from 40 pygmy camps located in southeastern Cameroon (Figure 1).

The sample consisted of 120 Baka pygmy children aged 5–6 years and 11–12 years selected according to non-exhaustive consecutive sampling. All children whose parents or legal guardians signed a consent form and who fell within the determined age ranges participated in the study.

Data collection was carried out by means of a four-part data sheet, the first of which included sociodemographic data, a second part consisting of a questionnaire on diet and oral hygiene practices, a third part on the various oral health services offered, and a fourth part consisting of a questionnaire on oral health services.

The oral examination was performed in natural light using instruments such as a flat mirror, periodontal probe (WHO community periodontal index probe), etc., according to the criteria recommended by the WHO. Within these recommendations, the diagnosis of caries is considered as an open caries lesion in dentin (equivalent to ICDAS IV or higher), which should be considered a limitation of the study.

The research team consisted of three people: the principal investigator responsible for conducting the clinical examination and the survey, an assistant student in the sixth year of oral medicine trained and qualified to fill in the data sheets, and finally a translator.

During the oral examination, an intra-examiner calibration was performed, and a high reproducibility and Kappa (K) consistency index (K above 85%) were achieved.

### 2.2. Study Variables

Sociodemographic variables such as age range, sex, Baka pygmy village or camp, and schooling level were used. Other variables including diet, oral hygiene habits, oral pathologies, and access to health services were evaluated.

### 2.3. Statistical Analysis

IBM SPSS Statistics 29.0 software (USA) was used. The Chi-2 test made it possible to evaluate the associations between variables with a significance level of *p* ˂ 0.05. The results for each variable are expressed as prevalence and percentage.

## 3. Results

### 3.1. Sociodemographic Data

The sample consisted of 120 Baka children consulted, of whom 69 were boys and 51 girls, with a sex ratio of 1.35. The 5–6 years age group was strongly represented (88.38%). Very few children (11.62%) were recorded in the 11–12 age group. Most of the population had CPC (Community Preschool Center) as their level of schooling (66.60%). CE1 (third primary) was the highest level of schooling (Table 1).

A total of 22 camps were visited during the study (Table 2).

### 3.2. Daily Diet and Oral Hygiene Habits

#### 3.2.1. Daily Diet

**Figure 2:**
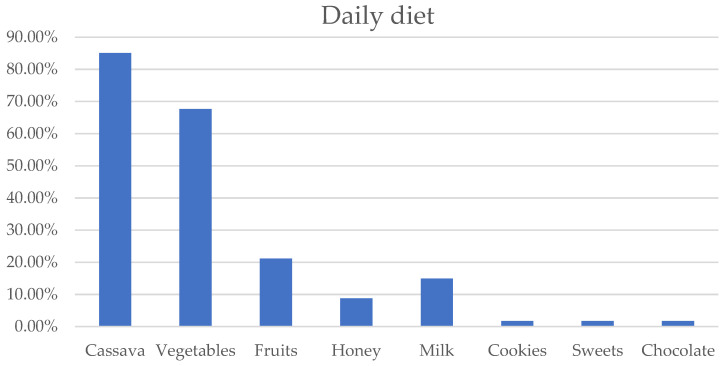
Distribution of daily diet in the overall study population.

Cassava (85.07%), vegetables (67.63%), and fruits (21.12%) were the most consumed foods. Cookies, sweets, and chocolate (2% each), which are more cariogenic, were less consumed (Figure 2).

#### 3.2.2. Oral Hygiene Habits

The identification of hygiene practices was based on whether or not the practices existed and their frequency, technique, instruments, aids, and time and frequency of brushing. In the population (n = 120), 69.17% (83/120) stated that they did not clean their teeth, of whom 60.24% (50/83) said they did not have a toothbrush, 21.69% (18/83) said they did not have enough time to brush their teeth, and 18.07% (15/83) said that they did not know how to brush their teeth.

In total, 37 of the 120 children assessed reported cleaning their teeth.

Very few children aged 5 to 6 years (3.7%) had an acceptable brushing frequency (twice to several times a day), whereas 39.4% [4] of the children aged 11 to 12 years had an acceptable brushing frequency (Table 3).

The brushing technique used by children in both age groups, i.e., 96% for the 5–6 year age group and 100% for the 11–12 year age group, was not adequate. Among the children who cleaned their teeth, using a toothbrush as the instrument and toothpaste as the main aid, the frequency was 81.48% in children aged 5–6 years and 100% in children aged 11–12 years. However, children aged 11–12 years also used soap and salt (40%) as an aid for the brushing (Table 3).

Overall, 63% of children aged 5–6 years had an inappropriate brushing time (morning before eating), while 50% of children aged 11–12 years had an acceptable brushing time (morning and afternoon).

The frequency of brush change was inappropriate for both age groups, i.e., 70% for the 5–6 years age group and 60% for the 11–12 years age group (Table 3).

### 3.3. Access to Oral Health Services

The survey on access to oral health services focused on the presence or absence of dental visits, location of visits, main speakers, type of intervention, frequency of visits, and last visit.

#### Dental Visits Received, Place of Visits, and Who Conducted Them

Only 13.70% (n = 17) of the study population reported having received a dental visit. The school was the place where they received the most dental visits (70.1%), and the main actors were NGOs (100%) (Table 4).

### 3.4. Interventions

Among the children who had received dental examinations (n = 17), 52.9% of the children in the 5–6 years age group and 67.5% in the 11–12 years age group reported receiving sealants and fluoridation as interventions (Table 5).

### 3.5. Frequency of Dental Visits

Most of the children in the 5–6 years age group reported having received a dental visit more than twice, i.e., 64.3%, and the same percetage (64.3%) of them in this age group reported having received their last dental visit more than one year ago (Table 6). Those in the 11–12 age group had received only one visit and that was exclusively more than one year ago. In addition, the supply of dental services is still insufficient.

### 3.6. Pathologies

#### 3.6.1. Periodontal Status

The gingival bleeding index as it is described in *Oral Health Surveys: Basic Methods* 5th edition [25] and the presence or absence of calculus were used to describe the periodontal status of the study population. Overall, more than 85% of the population had gingival bleeding, and less than half (30%) had dental calculus (Table 7). There was a statistically significant association between age and the presence of dental calculus (*p* = 0.018).

#### 3.6.2. Dental Caries

The parameters evaluated for dental caries were prevalence, the DMFT/dft index, and the restoration index.

Of the 2799 teeth examined, 2713 were caries-free, 85 teeth were decayed (D), only 1 tooth was missing (M), and no teeth were filled (F). The overall prevalence of dental caries was 27.5%. The dft in children aged 5–6 years was 0.735, and the DMFT in children aged 11–12 years was 0.553.

Despite a very low overall DMFT index (0.71 < 1.2), the restoration index representing the number of filled teeth in the population was zero, demonstrating the need for treatment for decayed teeth and the lack of dental treatment in this population.

#### 3.6.3. Other Oral Pathologies

Other diseases were evaluated in the study, and serious pathologies such as noma and necrotizing gingivitis (GN) were found (Table 8).

## 4. Discussion

### 4.1. Sociodemographic Data

The 5–6 years age group constituted 88.38% of the population studied. In the camps visited, the school structures found were mostly community preschool centers (CPCs), fully built and maintained by the NGO Zerca y Lejos since 2001 [26], which welcome and prepare Baka children from 2 to 7 years old for easier access to elementary school, unlike the 11- to 12-year-olds who are less present due to the absence of primary schools and also because at this age the children begin to participate in collection activities, gathering, and together with their parents are used as cheap labor in the field activities of the non-indigenous or Bantu groups [22], activities that keep them away from the camps. Of the population studied, 66.67% were still at the CPC level, reflecting a relatively low schooling rate because very few children were in primary classes. This analysis is close to that conducted by the United Nations in 2010 with respect to the indigenous population that noted a very low level of schooling, with only 1.31% of indigenous Baka children in the Salapoumbé region of eastern Cameroon attending elementary school [27].

### 4.2. Diet

Cassava, vegetables, fruits, and meat/fish were the main foods in the daily diet of the study population. Several studies describe the Baka pygmies as a forest-dwelling population characterized by a semi-nomadic way of life whose main activities are hunting, fishing, gathering, and, in recent years, agriculture for self-feeding [25,26]. However, these foods constitute the basis of the daily diet of the majority of the rural population in Cameroon [28,29]. In addition, only 1.74% of the population consumed sweet foods (cookies, sweets, chocolate) several times a week because of less accessibility to these foods due to the isolation of the camps.

### 4.3. Oral Hygiene Habits

The study showed that only 30.83% of the population reported cleaning their teeth. Higher proportions were found in a study conducted in rural areas of North West Cameroon in 2015 where 94% of the population reported brushing their teeth, which was explained by the fact that children would receive instructions from their parents regarding tooth brushing [30]. Of the 69.17% who said they did not brush their teeth, 60.24% had the reason of not having a toothbrush, 21.69% did not know how to clean their teeth, and 18.07% said they did not know how to brush. This reflects the conditions of misinformation and very low levels of education and economic income in which they find themselves [31]. As a brushing instrument, 100% of the population used a toothbrush. As an alternative to the toothbrush, 13.51% used their fingers. As an adjuvant, 86% of the population used toothpaste, 21.62% used soap, 19% used cooking salt, and 8% used charcoal. Considering the use of additives to brushing, these results are close to the study conducted by Azodo and Agbor in 2015 where 18.7% used soap, 20.2% used charcoal, and 50.2% used cooking salt [32]. A horizontal brushing technique was used by 90% of the population. A study conducted by V.Mots in 2011 reports different approaches to brushing technique, and the results show that horizontal brushing is the recommended one in primary teeth [33].

### 4.4. Access to Oral Health Services

Of the general population, 86.3% said they had never had a visit to the dentist. Only 13.7% said they had had a visit to the dentist, and 100% of them said they had received visits only from NGOs and in particular from the NGO Zerca y Lejos, which has been active in the region in the area of health, education, and defense of the rights of the Baka pygmy people since 2002 [26]. This highlights the almost total absence of public services for the provision of oral health services. It is also important to highlight the attachment of this population to their traditions and practices, distancing them from the knowledge and existence of conventional medicine [31,34]. In addition, the isolation of these areas and the reduced number of dental care personnel available for public services mean that these personnel are more concentrated in urban areas, which explains the scarcity or even non-existence of dental care services among this population [35]. According to the WHO report on global health statistics in 2013, per 10,000 inhabitants, Cameroon had a minimum rate of less than 0.05 dentists and an average of 2.2 dentists [36].

### 4.5. Pathologies

#### 4.5.1. Caries Prevalence and DMFT/dft

The overall prevalence of caries was 27.5%, and the overall DMFT/dft index was 0.71. A study conducted in 2012 on the distribution of the DMFT index in Africa reported an index of 1.70 for Cameroon [4]. In this study, there was no statistically significant relationship between the presence of caries and the foods consumed. However, this low prevalence rate observed is explained by the fact that the population studied was mainly in rural areas and that access to cariogenic foods (cookies, candies, chocolate, etc.) is limited or very low because only 1.74% of the population consumed this type of food. This result is very close to that found in a study conducted in rural areas of the Gulu region of Uganda (2016) which showed a prevalence of 27.1% and a DMFT index of 0.71 [37].

The prevalence of caries in the 5–6 years age group was 29.24% with a cod index of 0.735. Higher proportions of these results were found in a study conducted among indigenous children in Brazil in 2015, which showed a prevalence of 75% and a DMFT index of 3.11 [38]. This observed difference may be due to the specific dietary habits of each locality, since the accessibility of certain food products is not the same in all localities.

In the 11–12 years age group, the prevalence of caries was 14.28% and the DMFT was 0.553. A study conducted in Kabarole district in Uganda by Kutesa et al. in 2015 reported similar results with a prevalence of 27.1% and a DMFT of 0.48 in rural children aged 11–13 years [37].

Based on the criteria for the severity of carious disease in a population described by the WHO, a DMFT of less than 1.2 is considered very low and indicates a lower need for care for this population [39]. The one in this study is an example with a DMFT of 0.71. It is important to note that in this study the carious component (D) represented the essential part of this index, since out of the 2799 teeth studied we found 2713 healthy teeth, 85 decayed teeth, 1 missing tooth, and no filled teeth. The dental restoration rate, which is equal to the number of filled teeth out of the number of decayed and filled teeth or 0/85, was zero. The dental restoration rate, which is equal to the number of children with filled teeth out of the total number of children consulted, i.e., 0/120, was also zero. This shows that, despite reducing the need for treatment for carious teeth, a high probability of these teeth evolving to a complication stage in the absence of treatment can be observed.

These results highlight the need for much more preventive care. However, the current need for curative care for caries treatment is present despite an observed low prevalence.

#### 4.5.2. Periodontal Status

The periodontal status of the population studied showed the presence of bleeding on probing and dental calculus. Overall, 87% of the population had bleeding on probing (score 1), so the prevalence of gingivitis was 87%. This result is similar to that found in a study carried out in rural areas of Bafia and North West Cameroon, with a prevalence of 82% [19] and 89.6% [32], respectively, and could be explained by exposure to the same factors including the type of diet and especially the lack of practice of and motivation for oral and dental hygiene.

According to age, the proportions of dental calculus were 26.4% in children aged 5 to 6 years and 56.6% in those aged 11 to 12 years. This discrepancy observed could be explained in this study by the fact that, at the age of 11–12 years, children attending elementary school outside the camps could have changed their eating habits, as opposed to children aged 5–6 years who were found more in the community preschools present in the camps. Given these findings, there is a great need for periodontal care, including teaching and promoting oral hygiene practices as well as cleaning.

#### 4.5.3. Other Pathologies

Smoking, alcoholism, malnutrition, poverty, and poor oral hygiene are factors in the appearance of serious pathologies such as noma and necrotizing gingivitis (NG) or cancer [3,8,10], a context in which the population of this study is found, where 4.16% had NG and 1% had noma. However, the case of noma found in the study was in the process of being treated by the NGO Zerca y Lejos.

A study on the therapeutic itineraries of the Baka pygmies in southern Cameroon reveals that 5.8% of this population resorted to traditional therapists for the treatment of noma [34], so it would be of interest to assess and take into account these traditional perspectives and alternatives in the planning and adaptation of oral health in this context.

Overall, the results of the study showed that 62.5% of the population was affected by dental erosions. There was no statistically significant relationship between diet and the presence of dental erosion, but as hunter–gatherer populations a large part of their daily diet is based on the consumption of fruits (guavas, bananas, mangoes, pineapple, etc.) [31], which mainly contain acids that would be at the origin of these observed erosions.

Only 3.3% of the population had dental trauma, and no cases of dental mutilation were recorded. Dental mutilation is a traumatic act that has serious consequences for the teeth [21], and in the Baka context this act is part of a ritual process of initiation to puberty and beautification for adults [13], which could explain the non-existent rate of dental mutilation in our study.

## 5. Conclusions

The results show that oral diseases are present in the population, oral hygiene teaching observations are inadequate, and the supply of oral health care services for this population are almost non-existent and do not allow early and rapid intervention of oral pathologies in children. However, the population studied benefits from certain provisions of care coming exclusively from NGOs.

## Figures and Tables

**Table 1:** Distribution of sociodemographic data.

		Frequency	Percentage
Gender	Female	51	57.88%
	Male	69	42.51%
Age range	[5–6]	106	88.38%
	[11–12]	14	11.62%
Study level	CPC (Community Preschool Center)	80	66.60%
	SIL (1st grade)	17	14.40%
	CP (2nd elementary)	4	3.33%
	CE1(3rd grade)	5	4.18%
	CE2 (4th grade)	2	1.74%
	Non-schooling	12	9.75%

**Table 2:** Study population by camp.

Camp	Frequency	Percentage	Camp	Frequency	Percentage
Adjap Mintom	13	10.83%	Belleville	5	4.17%
Doum	12	10.00%	Opkweng	4	3.33%
Mfem	10	8.33%	Assock	4	3.33%
Akonetié	9	7.50%	Meyos Mintom	3	2.50%
Akom	8	6.67%	Ndibot	3	2.50%
Bemba2	8	6.67%	Bifilon	3	2.50%
Minkoo	7	5.83%	Mebane3	3	1.67%
Mveng	7	5.83%	Niabibete	3	1.67%
Zoulabot	7	5.83%	Mebane2	1	0.83%
Miata	6	5.00%	Mekas	-	-
Alouma	6	5.00%	Nkolasseck	-	-

**Table 3:** Distribution of toothbrushing tools and methods according to age range in the sample that reported brushing their teeth. The parenthesis indicates the *n* of the sample.

Age Range			Total
	[5–6] (n = 27)%	[11–12] (n = 10)%	(n = 37)%
**Toothbrushing frequency**			
3 times a day	(12) 44.4%	(3) 30%	(15) 40.5%
Twice a day	(1) 3.7%	(4) 40%	(5) 13.5%
Once a day	(4) 14.8%	(2) 20%	(6) 16.2%
Sometimes	(10) 37.0%	(1) 10%	(11) 29.7%
**Toothbrushing technique**			
Vertical	(1) 3.7%	-	(1) 2.7%
Horizontal	(26) 96.2%	(10) 100%	(26) 97.29%
Mixed	-	-	-
Circular	-	-	-
**Instruments used**			
Toothbrush	(27) 100%	(10) 100%	(37) 100%
Coal	-	-	-
Fingers	(1) 3.7%	(4) 40%	(5) 13.5%
Sticks	-	-	
**Toothbrushing aids**			
Dentifrice	(22) 81.48%	(10) 100%	(32) 91.4%
Soap	(4) 14.8%	(4) 40%	(8) 21.6%
Salt	(3) 11.1%	(4) 40%	(7) 19.0%
Coal/ashes	(1) 3.7%	(2) 20%	(3) 8.1%
Nothing	(21) 7.7%	(9) 90.0%	(30) 81.1

**Table 4:** Distribution of visits received.

		Frequency	Percentage
Visits (n = 120)	Visits received	17	13.7%
	No visits	103	86.3%
Place of visits (n = 17)	School	12	70.1%
	Camp	7	43.2%
	Hospital	-	-
Who conducted it? (n = 17)	NGO	17	100%
	Dentists of public services	-	-

**Table 5:** Distribution of interventions by age range and gender. The parenthesis indicates the n of the sample.

Interventions	[5–6] (n = 14)%	[11–12] (n = 3)%	Females (n = 9)%	Males (n = 8)%
Oral hygiene training	(3) 23.6%	(1) 33.3%	(3) 33.33%	(1) 12.5%
Tartrectomy	(1) 8.2%	-	(1) 11.11%	-
Sealants, fluoridation	(7) 52.9%	(2) 67.5%	(3) 33.33%	(6) 75%
Others (tooth extractions, etc.)	(3) 23.4%	-	(2) 22.22%	(1) 12.5%

**Table 6:** Distribution of dental visits according to gender and age range. The parenthesis indicates the n of the sample.

	Age Range		Gender	
	[5–6] (n = 14)%	[11–12] (n = 3)%	Females (n = 9)%	Males (n = 8)%
**Frequency of dental visits**				
Once	(5) 35.71%	(3) 100.0%	(6) 66.66%	(2) 25%
More than twice	(9) 64.3%	-	(3) 33.33%	(6) 75%
**Date of last visit**				
More than 6 months	(1) 7.14%	-	(1) 11.11%	-
More than one year	(9) 64.3%	(3) 100.0%	(8) 88.88%	(4)50%
I do not know	(4) 28.57%	-	-	(4) 50%

**Table 7:** Periodontal status according to age and gender in the study population. * *p* < 0.05 means statistical significance.

No Bleeding			Bleeding		Calculus		
Age range	Frequency	Percentage	Frequency	Percentage	Frequency	Percentage	*p* value
[5–6]	13	12.26%	93	87.74%	28	26.4%	
							0.018 *
[11–12]	2		12	85.7%	8	56.6%	
		14.3%					
Females	6	10.15%	45	76.27%	15	29.5%	
Gender							0.944
Males	9	14.75%	60	98.36%	21	30.1%	
TOTAL	15/120	13%	105/120	87%	36/120	30%	

**Table 8:** Distribution of other diseases in the study population. The parenthesis indicates the n of the sample.

	Gender			Age Range	
	Prevalence (n = 120)%	Males (n = 69)%	Females (n = 51)%	[5–6] (n = 106)%	[11–12] (n = 14)%
**Illness**					
Wounds, scars	(1) 0.83%	(1) 2.2%	-	(1) 2.2%	-
Noma	-	-	(1) 2.2%	(1) 1%	-
Tumors	(4) 3.9%	(4) 3.9%	-	(2) 3.0%	(2) 14.28%
Total	(5) 4.7%	(5) 7.2%	-	(3) 2.83%	(2) 14.28%
**Mucosal lesions**					
NG	(5) 4.16%	(3) 4.6%	(2) 4.1%	(4) 4.0%	(1) 7.1%
Abscess	(4) 3.33%	(3) 4.6%	(1) 2.0%	(4) 3.5%	-
Others (recessions)	(3) 2.5%	(3) 4.2%	-	(3) 2.8%	-
Total	(12) 10%	(9) 13.2%	(3) 6.1%	(11) 10.3%	(1) 7.1%
	(75) 62.5%	(47) 67.1%	(28) 56.2%	(72) 67.7%	(3) 22%
**Dental Mutilation**	-	-	-	-	-

## Data Availability

Data will be available from corresponding authors.
